# Clinical outcomes of anterior tibiofibular ligament’s distal fascicle transfer versus ligament reconstruction with InternalBrace™ for chronic ankle instability patients

**DOI:** 10.1007/s00402-021-04214-2

**Published:** 2021-11-30

**Authors:** Jiaxin Tian, Tsz-Ngai Mok, Tat-Hang Sin, Zhengang Zha, Xiaofei Zheng, Qiang Teng, Huige Hou

**Affiliations:** 1 Department of Sport Medicine, Institute of Orthopedics Diseases and Center for Joint Surgery and Sports Medicine, The First Affiliated Hospital of Jinan University, No. 613 Huangpudadao, Guangzhou, Guangdong China; 2 International School, Jinan University, GuangzhouGuangzhou, 510632 Guangdong China; 3 Department of Breast Surgery, Peking Union Medical College Hospital, Chinese Academy of Medical Science & Peking Union Medical College, Beijing, China

**Keywords:** Sports medicine, Ankle, Chronic ankle instability, Internal brace, Ankle arthroscopy

## Abstract

**Purpose:**

Treatment of chronic ankle instability (CAI) for ankle sprain patients remains a challenge. If initial treatments fail, surgical stabilization techniques including ligament reconstruction should be performed. Anterior tibiofibular ligament (ATiFL) distal fascicle transfer for CAI was recently introduced. The goal of the study is to assess the 1-year clinical effectiveness of ATiFL’s distal fascicle transfer versus ligament reconstruction with InternalBrace™ (Fa. Arthrex, Naples).

**Methods:**

Between October 2019 and February 2021, 25 patients (14 males and 11 females) scheduled for ligament reconstruction treatment of CAI were enrolled after propensity score matching. Twelve underwent ligament reconstruction with InternalBrace™ (InternalBrace™ group) and thirteen underwent ATiFL’s distal fascicle transfer (ATiFL’s distal fascicle transfer group). We recorded the American Orthopedic Foot & Ankle Society (AOFAS) score, Visual Analogue Scale (VAS), anterior drawer test grade, patient satisfaction and complications. All results of this study were retrospectively analyzed.

**Results:**

Statistically significant (*p* = 0.0251, independent-samples *t* test) differences in the AOFAS can be found between the ATiFL’s distal fascicle transfer group and the InternalBrace™ group. No substantial changes in the VAS (*p* = 0.1778, independent-samples *t* test), patient satisfaction (*p* = 0.1800, independent-samples *t* test) and anterior drawer test grade (*p* = 0.9600, independent-samples *t* test) were found between the two groups. There was one patient with superficial wound infection and one patient with sural nerve injury in the InternalBrace™ group and ATiFL’s distal fascicle transfer group, respectively.

**Conclusion:**

This is the first study that assessed a cohort of CAI patients and suggests that the ATiFL’s distal fascicle transfer operation has the potential to attain good-to-excellent clinical outcomes after 1-year recovery. The AOFAS scores were significantly higher for patients with ATiFL’s distal fascicle transfer, indicating that this technique may be considered a viable option for both patients and their surgeon, while long-term outcomes should be investigated in the future.

## Introduction

Chronic ankle instability (CAI) is a common pathological condition secondary to ankle sprains in professional athletes and the general population (1–5). Between 20 and 40% patients experience CAI ensuing from repeated sprains or inappropriate initial management of acute sprains (6). CAI is caused by functional or mechanical instability of lateral ankle ligaments, and people present with recurrent ankle instability events. The ligaments involved in CAI include the anterior talofibular ligament (ATFL), the calcaneofibular ligament (CFL) and the posterior talofibular ligament (PTFL) (7, 8). Moreover, intra-articulation lesions are found in 93% of patients who suffer from ankle instability (9). It is recognized that untreated CAI will lead to severe consequences including osteoarthritis, sybaritic and post-traumatic arthritis (10–14). In the clinic, conservative treatments include pain control, ankle activity restriction and physiotherapy (15). If initial treatments fail, surgical options such as anatomic repair, ligament reconstruction and augmented repair should be considered using either arthroscopy or open surgery (3, 16).

At present, a new method for CAI patients who cannot undergo a direct repair operation is ligament reconstruction. The graft types, fixation materials and methods vary widely in different ligament reconstruction procedures and have been described in previous studies (17–23). In many operations, InternalBrace™ (Fa. Arthrex, Naples) is used to strengthen the CAI-related ligaments (10). InternalBrace™ reconstruction allows residual ligament tissue to acquire additional strength. Excellent long-outcomes for reconstruction had been proved especially for patients with poor tissue quality or failure of the previous repair operation, and young patients with sports needs (24, 25). Unfortunately, the relative difficulty of the techniques, the significantly high cost of procedures and other complications were noticed by many surgeons. Currently, some researchers propose that the anterior tibiofibular ligament’s (ATiFL) distal fascicle, which also known as Bassett’s ligament (18) can be transferred as a reconstruction technique to treat CAI (22, 26). Some studies (27–29) have declared that ATiFL’s distal fascicle played an important role in ankle function and could be used as a safer and reliable biological reinforcement for the ATFL repair (26).

There is controversy surrounding which method should be chosen during chronic ankle instability operation. Studies on ATiFL’s distal fascicle transfer are limited. Current studies only focus on the outcomes of anatomic study (26), while clinical outcomes are seldom mentioned. The purpose of this study is to compare the clinical outcomes of ATiFL’s distal fascicle transfer technique and ligament reconstruction with the InternalBrace™ technique in treating CAI patients using the AOFAS score, the VAS score, the anterior drawer test and a patient satisfaction score.

## Materials and methods

The trial was a single center, retrospective trial with the primary objective to evaluate the hypothesis that ATiFL’s distal fascicle transfer operation (minimally invasive surgery) was not inferior to ligament reconstruction with InternalBrace™. Information was collected from all participants after preoperatively obtaining written informed consent in accordance with the Declaration of Helsinki. At the beginning, 29 traceable patients from all ages in the general population treated in our hospital were enrolled (13 underwent ATiFL’s distal fascicle transfer operation and 16 underwent ligament reconstruction with InternalBraceTM) from October 2019–February 2021. There was no randomization between ATiFL’s distal fascicle transfer and ligament reconstruction surgery groups, and which technique to use was determined by one orthopedic senior surgeon who specializes in treating athletic injuries of ankle with arthroscopic (minimally invasive) procedures. Then, propensity score matching (PSM) was applied to achieve balanced groups at baseline using a logistic regression model. Final covariates were age, sex, preoperative AOFAS scores, preoperative VAS scores and preoperative anterior drawer test scores. The matching ratio is 1:1 with standard caliper width of 0.05. It is was approved by the ethics committee of the First Affiliated Hospital of Jinan University.

### Inclusion and exclusion criteria

For this investigation, clinical and stress radiological examinations were utilized together to define CAI. Inclusion criteria were: patients with more than one episode of ankle instability or ankle sprains within 6 months; the grades of ankle mechanical laxity for patients were more than one on the clinical anterior drawer test; patients with differences between two ankle laxity of 10 degrees in talar tilt angle or absolute talar tilt angle of 15 degrees during radiographic evaluation. To exclude interference factors of our study, the patients with insufficiency of ATiFL’s distal fascicle were eliminated. In addition, according to the radiographic classification (30), patients who suffered from CAI combined with rheumatoid arthritis or grade II or greater of ankle degenerative arthritis were not considered in this study. Other factors that would influence outcomes were also excluded (i.e., systemic disease history, neuromuscular disorder history, obesity). The eligibility criteria are listed in Table 1.

**Table 1:** Eligibility criteria applied in this study

Inclusion criteria	Patients with more than one episode of ankle instability or ankle sprains within 6 months. The grades of ankle mechanical laxity for patients ≥ 1 on the clinical anterior drawer test Patients with differences between two ankle laxity of 10 degrees in talar tilt angle or absolute talar tilt angle of 15 degrees during radiographic evaluation
Exclusion criteria	The patients with insufficiency of ATiFL’s distal fascicle According to the radiographic classification, patients who suffered from CAI combined with rheumatoid arthritis or grade II or greater ankle degenerative arthritis Other factors: systemic disease history, neuromuscular disorders history, obesity, etc.

**Table 2:** Baseline characteristics

Variable	Group InternalBrace™	Group ATiFL’s distal fascicle transfer	*P* value
Number of patients	12	13	
Age, years	32.82 ± 5.67	33.29 ± 4.93	0.342
Sex (M/F), *n*	6/6	8/5	
Follow-up, months	12.31 ± 2.02	12.31 ± 2.25	0.995
AOFAS	68.49 ± 8.55	68.46 ± 7.70	0.990
VAS	6.20 ± 1.05	5.77 ± 1.48	0.400
*Anterior drawer test, *%			0.8408
Grade 0	0%	0%	
Grade 1	3 (25.00%)	2 (15.38%)	
Grade 2	3 (25.00%)	5 (38.45%)	
Grade 3	6 (50.00%)	6 (37.5%)	

Data are number of patients *n* (%) or mean ± SD

*ATiFL* anterior tibiofibular ligament, *AOFAS* the American Orthopedic Foot & Ankle Society score, *VAS* visual analog scale for pain score

### Surgical technique

#### ATiFL’s distal fascicle transfer technique

General anesthesia was administered and the lateral decubitus position with ankle dorsiflex was used when performing this surgery.

**Fig. 1:**
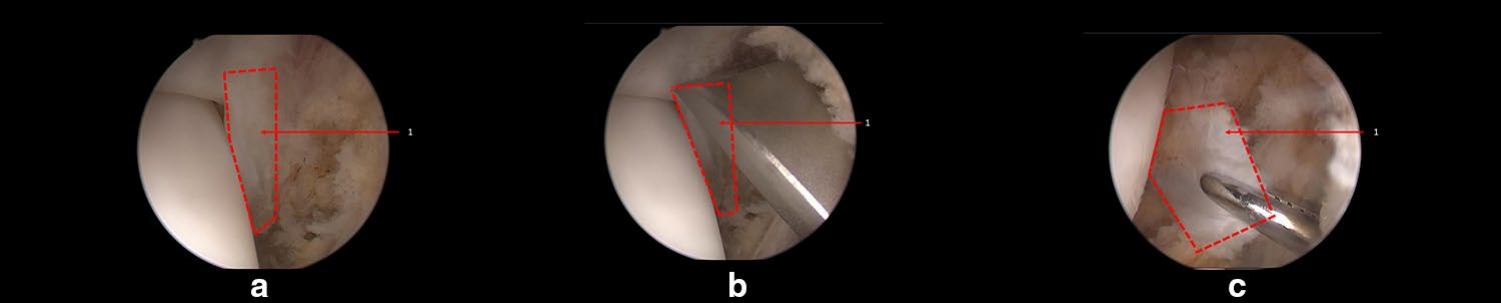
Structures in the lateral ankle region. **a** Exposure of the anterior tibiofibular ligament. **b** Stripping the anterior tibiofibular ligament. **c** Extraction of the anterior tibiofibular ligament. *(1) red zone: Anterior Tibiofibular Ligament

Arthroscopy examination was performed to observe a number of structures including anterior tibiofibular ligament in the lateral ankle region (Fig. 1). First, anteromedial portal, proximal anterolateral portal and distal anterolateral portal were carefully created without damage to the superficial peroneal nerve.

The ATiFL’s distal fascicle transfer was performed after the arthroscopic examination. Under arthroscopic guidance, a suture passer was inserted through the proximal anterolateral portal to grasp the ATiFL’s distal fascicle and penetrated it from proximal to the distal. Next, an arthroscopic grasper pulled out the Nitinol loop wire, which was changed by a folded-in-half FiberWire suture through the distal anterolateral portal. After this suture was pulled back, another double suture was firmly performed from the distal anterolateral portal to the proximal anterolateral portal with ATiFL’s distal fascicle in the center. The suture limbs that existed in the proximal anterolateral portal were pulled out through the distal anterolateral portal and passed through the suture loop. ATiFL’s distal fascia was firmly grasped by pulling the suture limbs.

**Fig. 2:**
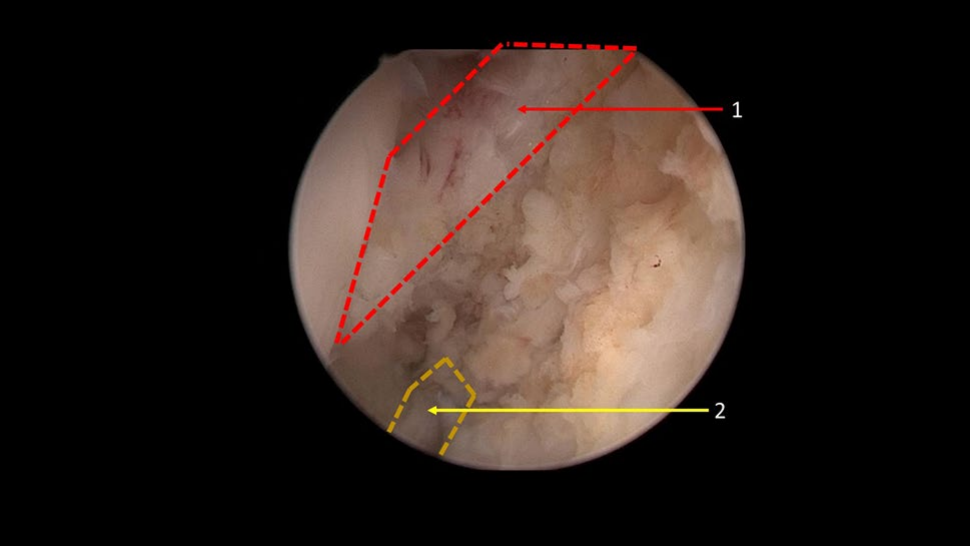
Anterior tibiofibular ligament’s distal fascicle is detached. (1) Anterior Tibiofibular Ligament. (2) Lateral Ligament Repair

Osteotome was then introduced for the detachment of ATiFL’s distal fascicle via the proximal anterolateral portal. The whole structure (tibial origin of the ligament and its small bony fragment) was moved to the ATFL’s talar insertion (Fig. 2).

**Fig. 3:**
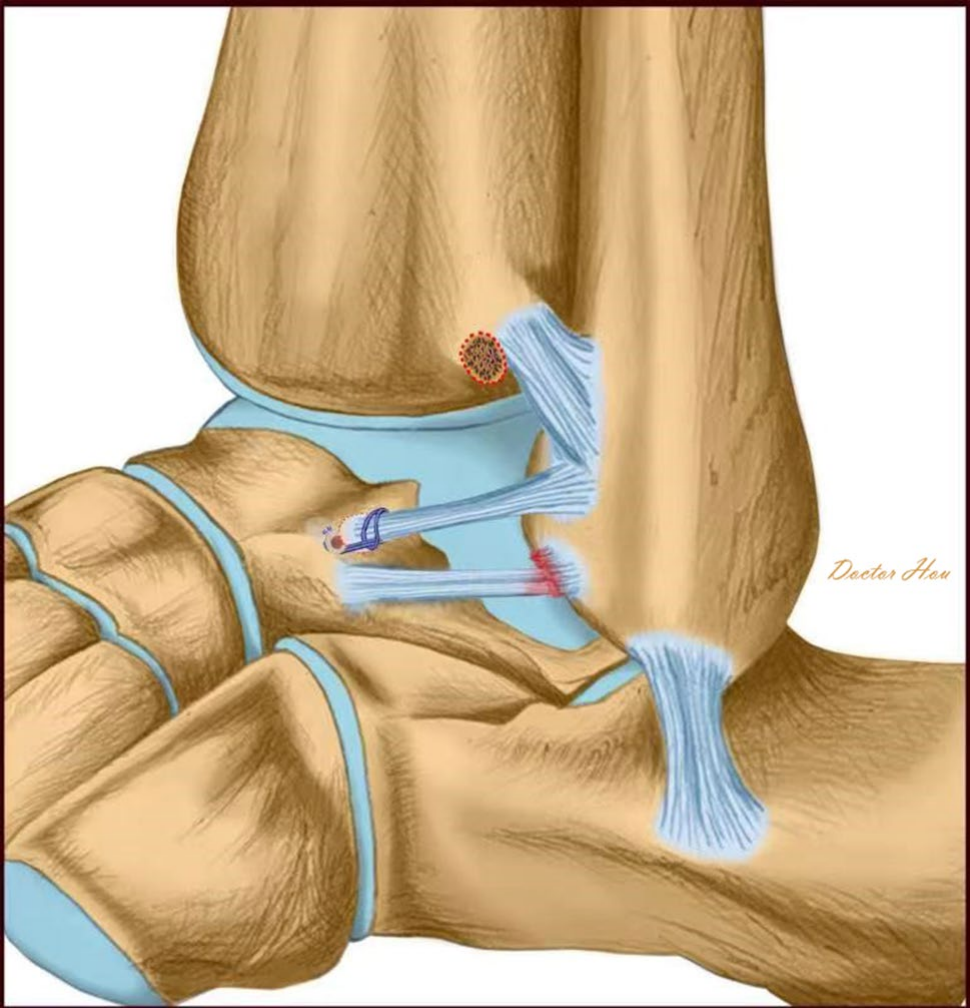
Schematic plot after the tibial origin of the ligament and its small bony fragment was moved to the ATFL’s talar insertion with one anchor at the distal side

To fit a talar tunnel, a talar bed was created because of the insufficiency of the transferred ligament’s length. A knotless bone anchor was introduced anterior to the talar bed on the talar neck to fix the ATiFL’s distal fascicle to its new location. The drill was used next. Beginning at the distal anterolateral portal, the hole was held in position directly pointing to the medial malleolus tip using a drill guide. The bone anchor and the suture in it were then placed into the hole by impaction. The schematic plot after surgery is shown in Fig. 3.

#### Ligament reconstruction with InternalBrace™

In our ligament reconstruction with InternalBrace™, general anesthesia and arthroscopic procedure were also utilized before initiating the procedure. The ankle joint was distracted with the help of a noninvasive distractor. Then, similar anteromedial and anterolateral portals were carefully created without damage of the superficial peroneal nerve. The anchor was placed in the distal anterior fibula through the anterolateral portal.

Two anchors were then introduced with a mallet into the talus grooves after removing the fibrocartilage on the talus and subchondral bone. The second anchor was seated into the fibula at the same level as the lateral shoulder of the talus.

The next step was suture tape augmentation for internal bracing. Using a calibrated drill guide with a tap, a tunnel was created between two anchors that we seated before. The suture tape, composed of ultra-high-molecular weight polyethylene and polyester, combined with a suture anchor were seated into the fibula. Then, this suture set was tensioned by surgical knots, and the remnants of the suture were not cut. The limbs of the suture were passed through the accessory portal and anterolateral portal in turn. Both bone anchor and suture were introduced into the hole. Finally, the suture remnants were cut off.

#### The postoperative process

After the operation, the ankles of patients in both groups were immobilized in a neutral position with a short leg cast for 2 months. No weight-bearing was allowed until the cast was removed from the ankle. After the cast was no longer required, progressive weight-bearing was allowed until all weight-bearing ability was recovered. Formal physical therapies, including proprioceptive training and eversion exercises, were initiated. A full range of sports was allowed at 3 months.

#### Clinical assessment

Postoperatively, the clinical assessment was performed by one orthopedic senior surgeon who did not partake in any surgical procedure or acknowledge any participant in this trial. Patients returned at 1, 2, 6, 12 and 16 months after surgery and recorded clinical outcomes at the final follow-up. The mean postoperative follow-up duration was 14 months (12–16 months) (27). The American Orthopedic Foot & Ankle Society (AOFAS) score was selected as the outcome measurement for assessing the functional status, considering 90–100 points as “excellent”; 80–89 points as “good”; 60–79 points as “fair”; and less than 60 points as “poor” (31, 32). The anterior drawer test was used as the criteria for ankle instability evaluation. Four levels were used to classify ankle instability. Normal (grade 0) was less than 5 mm translation compared with the opposite side, grade 1 was 5–10 mm side-to-side difference, grade 2 was 10–15 mm side-to-side difference, and grade 3 was more than 15 mm difference. The current pain levels of patients were rated on a 10-point visual analog scale (VAS) with 0 indicate to no pain and 10 to indicate very severe pain (33).

Complications including hematoma requiring surgery, surgical site infection, and superficial fibular nerve or sural nerve injury (34) were sought routinely and recorded as another outcome measurement for evaluating this ankle surgery.

Overall satisfaction of patients’ surgical results was also collected by asking patients to fill out satisfaction questionnaires. Results were recorded from 0 (dissatisfied) to 10 (very satisfied) (34).

### Statistical analysis

R, version 2.14.2 (R Development Core Team, Vienna, Austria) was used for statistical analysis. Paired data analysis correlated with the clinical evaluation was performed to compare between the two groups. T test was used to compare the results of AOFAS score, VAS score, satisfaction rate and anterior drawer test score with statistical significance established at ƿ < 0.05.

## Result

**Fig. 4:**
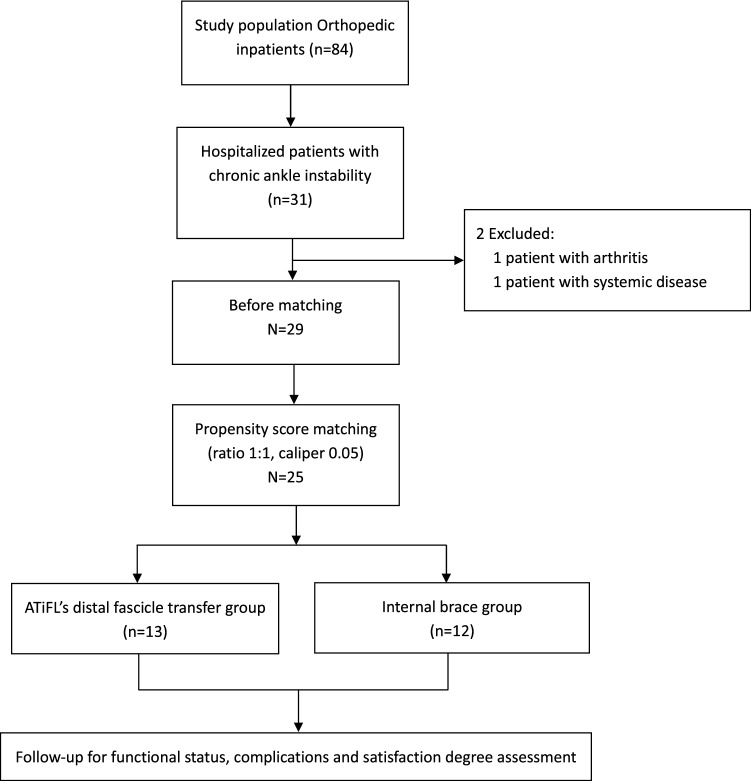
Trial profile

From October 2019 through February 2020, a total of 25 patients (14 males and 11 females) were enrolled after PSM (Fig. 4). Among them, 12 patients including 6 women and 6 men underwent ligament reconstruction with InternalBrace™. The remaining 13 patients, including 5 women and 8 men, underwent ATiFL’s distal fascicle transfer reconstruction. After propensity score matching, the two groups were comparable. Patients’ median age at surgery was 33.2 years (range 22–40). The two groups had no significant difference with respect to gender, follow-up duration, preoperative AOFAS score, preoperative VAS score and preoperative anterior drawer test grade. A comparison of baseline characteristics in both groups is displayed in Table 2.

The mean postoperative VAS score on a 0–10 scale was 4.01 ± 1.37 in InternalBrace™ group and 3.31 ± 1.32 in the ATiFL’s distal fascicle transfer group. No substantial differences were seen in the VAS score (*P* = 0.178, independent-samples *t* test) (Table 2). The mean patient satisfaction score was 7.27 ± 1.29 and 7.92 ± 1.32 in the InternalBrace™ group and the ATiFL’s distal fascicle transfer group, respectively. No significant differences were found in patient satisfaction score (*p* = 0.180, independent-samples *t* test) (Table 3). There was also no significant variation between the two groups considering the anterior drawer test (*p* = 0.960, independent-samples *t* test). Overall, there were 8 (66.7%) patients with grade 0 (normal) laxity and 4 (33.34%) patients with grade 1 laxity in the internal brace group. There were 9 (69.23%) patients with grade 0 (normal) laxity, 3 (23.08%) patients with grade 1 laxity and 1 (7.69%) patient with grade 2 laxity in the ATiFL’s distal fascicle transfer group (Table 3).

**Table 3:** Outcome characteristics

Variable	Group	Group	*P* value
	InternalBrace™	ATiFL’s distal fascicle transfer	
AOFAS	86.45 ± 4.08	89.85 ± 3.65	0.025
VAS	4.01 ± 1.37	3.31 ± 1.32	0.178
Satisfaction	7.27 ± 1.29	7.92 ± 1.32	0.180
*Anterior drawer test*, %			0.960
Grade 0	8 (62.50%)	9 (69.23%)	
Grade 1	4 (37.50%)	3 (23.08%)	
Grade 2	0 (0%)	1 (7.69%)	
Grade 3	0(0%)	0(0%)	

Data are number of patients *n *(%) or mean ± SD

*ATiFL* anterior tibiofibular ligament, *AOFAS* the American Orthopedic Foot & Ankle Society score, *VAS* visual analog scale for pain score

However, the between-group difference in AOFAS score was significant (*p* = 0.025, independent-sample *t* test). The AOFAS score was considered “good” (86.45 ± 4.08) in the InternalBrace™ group and “excellent” (89.85 ± 3.65) in the ATiFL’s distal fascicle group (Table 3).

Two patients engaged in our research suffered from complications. One in the InternalBrace™ group developed superficial infections around the operative region, which were controlled successfully with oral antibiotics. One in the ATiFL’s distal fascicle transfer group was found having sural nerve injury.

## Discussion

The most important contribution of this study is that for the first time, the clinical improvement and functional outcome of ATiFL’s distal fascicle transfer operation was assessed compared with ligament reconstruction surgery with InternalBrace™ in CAI patients in a controlled trial.

**Fig. 5:**
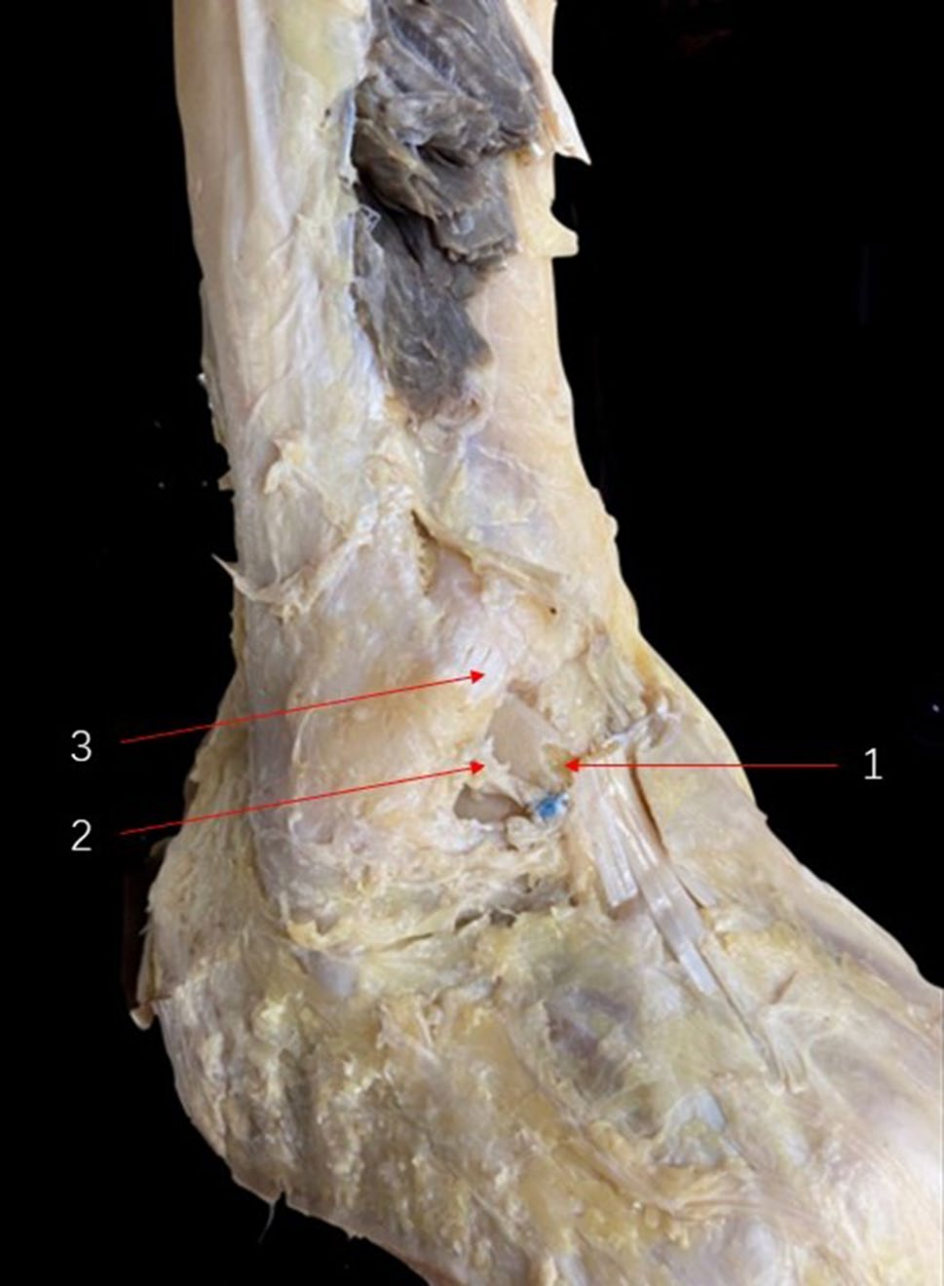
Lateral view of osteoarticular dissection after an anterior tibiofibular ligament’s distal fascicle transfer. (1) Bare area of the talus. (2) Anterior tibiofibular ligament’s distal fascicle transferred to the talar neck. (3) Anterior tibiofibular ligament

Many iterations of repair and reconstruction surgeries were described and evaluated, including the modified Brostrom operation (MBO)(4), arthroscopic modified Brostrom operation with a nonabsorbable InternalBrace™ (27), reconstruction with semitendinosus autografts (35), and anatomical reconstruction of ligament with a gracilis ligament graft (36), Chrisman–Snook operation for reconstruction of lateral ligament (37). Although these previous surgical procedures have demonstrated excellent results, some disadvantages, including donor site morbidity associating with autograft harvesting, significant high cost of procedures and immunogenic response associating with allografts, were reported. Because the relationship between ATiFL’s distal fascicle and ankle anterolateral soft-tissue impingement was proposed by some researchers, the possibility of utilizing this ligament as a biological reinforcement to treat CAI have been put forward in some studies. An anatomical study (26) was previously performed and showed excellent outcomes for anatomical reconstruction. These favorable outcomes were proved by our repeat anatomic study (Fig. 5). It is also noteworthy that the ligament was sutured in the previous anatomical study, which may have caused ligament damage during a real operation. To avoid this limitation, we designed a loop ligature to prevent ligament damage. In our study, clinical outcomes were evaluated and good-to-excellent functional outcomes, pain control and complication control in ATiFL’s distal fascicle transfer group were observed.

In our study, symptomatic patients who used ATiFL’s distal fascicle as a biological reinforcement for an ATFL reconstruction presented statistically significant improvement of AOFAS score from before surgery to final follow-up (ƿ < 0.05). These findings may be supported by the hypothesis that ATiFL’s distal fascicle can retain the ankle’s normal receptor population (26). Some neuroanatomical studies on the ATiFL’s distal fascicle have shown that using this ligament as biological reinforcement has predominance of type IV mechanoreceptors (37, 38), which relate to pain sensation, and type II mechanoreceptors (39), which relate to acceleration or deceleration of the joint.

Paresthesia and neurological complication evaluations were also performed in this study. Overall, both methods showed excellent outcomes in complication rates. Our results also showed no substantial difference in anterior drawer tests after recovery from the operation. Despite the favorable outcomes, we acknowledge that one individual who underwent ATiFL’s distal fascicle transfer operation suffered from grade 2 laxity. As this patient went back to work immediately after the operation, inappropriate postoperative rehabilitation may be closely related to this condition. There was no statistical variation in VAS score between two groups. Overall, the functional and clinical results indicated that using ATiFL’s distal fascicle as reinforcement for the ATFL reconstruction was objectively successful.

In addition, after comparing the satisfaction of patients who underwent ATiFL’s distal fascicle transfer operation with those underwent ligament reconstruction with InternalBrace™ operation, similarly high-degree satisfaction rates were recorded at the final follow-up. Despite these high-degree satisfaction rates from patients, the long operation time was mentioned by some surgeons who performed new technique. During the operation, surgeons spent much time stripping the ligament, which is a new technique that was suggested in 2018, and therefore, surgeons would have a learning curve to master the technique. Overall, considering that occasional pathological change in this ligament was found in the prior study, which may contribute to tibiotalar impingement syndrome, resection of this ligament for lateral ligament reconstruction was considered a valid and reliable method for treating CAI.

The use of ATiFL’s distal fascicle as a biological reinforcement also economical and practical. During ATiFL’s distal fascicle transfer, only one anchor was inserted in the distal part, and no anchor was needed in proximal part. Therefore, the fees of the operation were decreased significantly. Because different patients have different widths and lengths of ATiFL’s distal fascicle, therapeutic methods should be chosen according to individual patients.

It is important to recognize the limitations of this study. First, because it uses a new technique, the minimum follow-up of 12 months and mean follow-up of 14 months are too short to investigate the long-term functional outcome. Good-to-excellent patient-oriented outcome and clinician-oriented outcome with 1-year follow-up were recorded in our study, while longer term cohort studies are needed after patients return to sports. A second limitation of the study that warrants discussion is that a relatively small number of patients were assigned to ATiFL’s distal fascicle transfer operation, and thus, additional multi-center controlled trials are required to corroborate these findings. However, we would such as to report these promising results of our new technique for treating CAI. Based on these results, we firmly believe that chronic ankle instability can be successfully treated when primary repairment is not available. Time should be given for learning and evaluating this technique in further studies.

## Conclusion

In conclusion, ATiFL’s distal fascicle transfer operation seems to provide similar complication rates and patient satisfaction rates as ligament reconstruction with the InternalBrace™ operation. Our initial data indicate that the new method has potential to attain good-to-excellent clinical outcomes. At the same time, this new method has been confirmed to be both economical and practical. We believe that this technique may be considered a viable treatment option for chronic lateral ankle instability for both patients and surgeons.

## Abbreviations

AOFAS: American orthopedic foot & ankle society
ATiFL: Anterior tibiofibular ligament
CAI: Chronic ankle instability
VAS: Visual analogue scale
